# Rituximab in Antimelanoma Differentiation-Associated Protein-5 Dermatomyositis with Interstitial Lung Disease

**DOI:** 10.1155/2020/8145790

**Published:** 2020-05-30

**Authors:** Chiara Scirocco, Andreina Gubbiotti, Alfredo Sebastiani, Gian Domenico Sebastiani

**Affiliations:** ^1^UOC di Reumatologia, Azienda Ospedaliera San Camillo–Forlanini, Rome, Italy; ^2^UOC di Patologia Clinica, Azienda Ospedaliera San Camillo–Forlanini, Rome, Italy; ^3^UO Delle Interstiziopatie Polmonari, Azienda Ospedaliera San Camillo–Forlanini, Rome, Italy

## Abstract

In this paper, we report a challenging case of a middle-age woman who developed antimelanoma differentiation-associated protein-5 dermatomyositis (anti-MDA5 DM) with interstitial lung disease (ILD) and was successfully treated with rituximab (RTX), after failure of a first-line therapy.

## 1. Introduction

Idiopathic inflammatory myopathies (IIMs) are rare, chronic autoimmune diseases that affect primarily the skeletal muscle but can involve multiple organs; they can be isolated or represent a secondary manifestation of cancer or a previous autoimmune condition [1]. IIMs include different clinical subtypes of diseases such as dermatomyositis (DM), polymyositis (PM), immune-mediated necrotising myopathy (IMNM), overlap myositis (OM), and inclusion body myositis (IBM) [2, 3].

Autoantibodies in IIMs are divided into two different groups: myositis-associated autoantibodies (MAAs), more prevalent in overlap syndromes, and myositis-specific autoantibodies (MSAs). MSAs represent not only a powerful diagnostic biomarker, but they also have an important role in the pathogenetic, phenotypical, and prognostic characterization of the disease [4]. Antisynthetase syndrome (ASSD), the most common within OM, is characterised by a clinical triad represented by myositis, arthritis, and interstitial lung disease (ILD) and positivity of specific autoantibodies that are addressed to different aminoacyl-tRNA synthetases (ARS) [5, 6]. Disease course of IIMs shows a wide variability in different patients [7]. Among others, DM may involve the muscles, skin, and lung with various degrees of severity. In some cases, there is a prevalent skin disease but minimal or absent muscle one, and myopathy is defined as amyopathic DM (CADM) [8]. Specific autoantibodies are present only in 50–70% of patients with DM and CADM [9]. Antimelanoma differentiation-associated protein 5 (anti-MDA5) myositis is a specific subset of DM characterised by anti-MDA5 autoantibody positivity, a specific cutaneous phenotype with tender red or purple papules on the dorsal aspect of metacarpophalangeal and interphalangeal joints and/or elbows (Gottron's papules), development of acute, often severe ILD associated with mild or absent muscle involvement, and, frequently, arthritis and weight loss [10, 11]. Current management of IIMs is based on glucocorticoids, the mainstay of the treatment, and immunosuppressive agents, such as azatioprine, cyclosporine, mycophenolate mophetil, and cyclophosphamide. Moreover, escalation to rituximab (RTX), a chimeric monoclonal antibody for depleting B cells showing CD20 protein, can be evaluated in refractory forms [3, 12]. Particularly, RTX seems effective for the treatment of the skin and lung involvement in IIMs [13–20]. Few case reports addressing the use of RTX in anti-MDA5 DM and associated ILD patients have been described in the literature to date [18–20].

We herein report the case of a middle-age woman who presented cutaneous peculiar lesions, muscle weakness, ILD, and anti-MDA5 positivity and was successfully treated with RTX, after failure of a first-line therapy.

## 2. Case Presentation

On December 2017, a 50-year-old Caucasian woman, previously in good health, developed cutaneous erythematosus papular lesions both on the palmar and dorsal surface of hands and fissuring of the distal fingers, in the absence of other symptoms. She consulted a dermatologist who prescribed topical steroids. Three months later, Raynaud's phenomenon and dryness of the skin appeared. She consulted a rheumatologist who, after laboratory examinations showing antinuclear antibody (ANA) positivity, made the diagnosis of undifferentiated connective tissue disease (UCTD) and prescribed prednisone 25 mg daily with progressive tapering until 12.5 mg daily, with partial improvement. Two months later, she developed arthritis of hands and wrists, alopecia, and worsening of the skin lesions. She consulted another rheumatologist who added hydroxychloroquine 400 mg daily without any improvement. Instead, asthenia, muscle weakness, and pain appeared. For this reason, the rheumatologist prescribed cyclosporine 160 mg daily; hydroxychloroquine was stopped because of the onset of vertigo, which disappeared with drug suspension, while prednisone 12.5 mg daily was continued. On October 2018, because of further worsening of asthenia and myalgia and the onset of mild dyspnea, high-resolution computed tomography (HRCT) of the chest was performed, and it showed initial signs of ILD.

Then, the patient was referred to our attention and she was admitted to our rheumatology ward.

**Figure 1:**
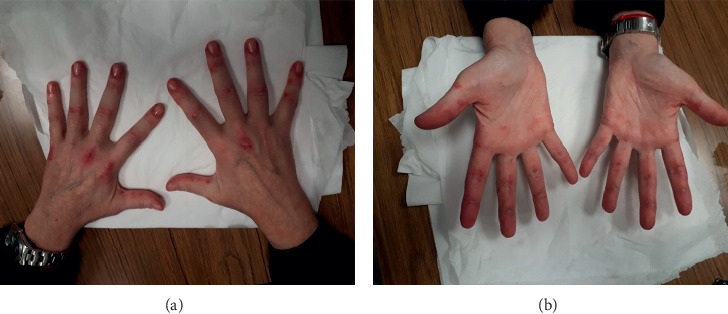
Papular erythematosus lesions on both the palmar and dorsal surface of the patient's hands.

Concerning the medical history, she did not report comorbidities, pregnancies, or miscarriages. She had smoked for ten years, but she was not smoking anymore. She was in menopause from the age of 45. Her family history was unremarkable. At the physical examination, she was afebrile, normotensive, and breathing and cardiac rates were, respectively, 16 times/min and 90 beats/min. Cardiac and abdominal examinations were normal; pulmonary examination showed fine bilateral basal crackles. There were papular erythematosus lesions on both the palmar and dorsal surface of her hands and extensor surface of elbows and concomitant fissuring of the distal fingers (Figure 1). Musculoskeletal examination did not evidence joint tenderness/swelling or deformity, but it revealed a reduction of muscle strength at the four limbs. Laboratory tests showed a mild normochromic, normocytic anemia, a slight increase of aspartate aminotransferase (AST) (52 U/L; normal range <40 U/L) and of lactate dehydrogenase (LDH) (688 U/L; normal range <400 U/L), a modest polyclonal hypergammaglobulinemia, and an increase of C reactive protein (CRP) (10 mg/L; normal range <5 mg/L), while white blood count, platelets, creatinine, electrolytes, erythrocyte sedimentation rate (ESR), uric acid, alanine aminotransferase (ALT), creatine phosphokinase, and urinalysis were normal. Screening for hepatitis B and C virus was negative, while the QuantiFERON-TB Gold test was indeterminate.

A skin right hand biopsy revealed the presence of interface dermatitis, with perivascular inflammatory infiltrate prevalently at the dermoepidermal junction and injury of the basal cells keratinocytes, as it can be observed either in systemic lupus erythematosus (SLE) or in DM [21].

Electromyography (EMG) of the upper limbs evidenced signs of median neuropathy in the right hand and no other pathological sign.

**Figure 2:**
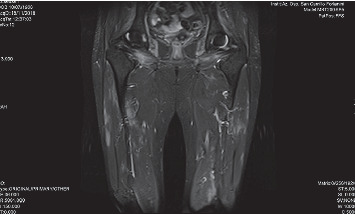
Evidence of diffuse and symmetric muscles oedema of all the muscles of the thigh at MRI.

Muscle magnetic resonance (MRI) of the lower limbs showed diffuse and symmetric oedema of all the muscles of the thigh without fatty degeneration (Figure 2).

Chest HRCT confirmed the presence of bilateral apical pleural thickening and various subpleural, paramediastinal, and basal areas of reticular thickening associated with ground glass lesions. There was no pleural effusion or linfoadenopathy. Spirometry with diffusing capacity for carbon monoxide (DLco) evidenced signs of a disventilatory obstructive syndrome with severe DLco reduction (53% of the predicted value). Echocardiogram was normal.

**Figure 3:**
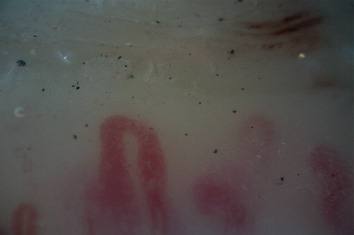
A nailfold capillaroscopy that shows alterations indicating a “scleroderma-like pattern.”

A nailfold capillaroscopy revealed different alterations such as a disordered loops architecture, capillary enlargement with some megacapillaries, microhaemorrhages, and neoangiogenesis, suggestive of a “scleroderma-like pattern” (Figure 3).

Indirect immunofluorescence (IIF) for ANA showed a cytoplasmic pattern at a titer of 1 : 160; rheumatoid factor, anti-SSA (Ro), anti-SSB (La), anti-Sm, anti-RNP, anti-Jo1, anti-SCL-70, anti-ds DNA, C3, C4, and anti-citrullinated peptide antibody (ACPA) were normal, while a myositis panel immunoblotting test evidenced anti-MDA5 positivity (53 AU; normal range <10).

Therefore, the patient was discharged with a diagnosis of anti-MDA5 DM and ILD. Prescribed therapy was mycophenolate mofetil 2 gm daily and prednisone 25 mg daily associated with a weekly bisphosphonate tablet and a prophylactic therapy for *Mycobacterium tuberculosis*.

At the follow-up visit, two months later, the patient reported worsening of fatigue, weakness, and dyspnea. Her laboratory exams showed an increase of inflammatory indexes (ESR 50; normal range <30; CRP 15 mg/L; normal range <5 mg/L), while a spirometry evidenced a further DLco reduction (42% of the predicted value).

After consultation with the respiratory physician, we decided to discontinue mycophenolate and start a second-line therapy with RTX 1000 mg on two occasions 2 weeks apart. Prednisone was continued with progressive tapering to 12.5 mg daily.

Patient was re-evaluated two months later. She reported a marked improvement of fatigue, muscle weakness, and dyspnea. At physical examination, the skin lesions had improved. Laboratory tests showed a reduction of ESR and CRP values (ESR 30; normal range <30; CRP 5; normal range <5 mg/L). DLco parameters were significantly ameliorated (from 42% to 56% of the predicted value). Therefore, it was possible to reduce prednisone dosage from 12.5 mg to 10 mg daily.

The patient is currently under follow-up in our unit.

## 3. Discussion and Conclusions

Anti-MDA5 myositis is a specific subset of DM characterised by the presence of anti-MDA5 autoantibodies and a peculiar cutaneous phenotype with ulcers and vasculopathy, mild or absent muscle involvement, severe ILD, and high mortality rate [10, 11].

MDA-5 belongs to the family of retinoic acid-inducible gene I-(RIG-I-) like receptors (RLRs), and it is involved in the innate immune response to viral infections [22]. Anti-MDA5, the specific autoantibody against this target autoantigen, has a pathogenetic role in the development of the disease and also a prognostic role; its circulating levels may predict the response to therapy and the probability of relapse [23, 24]. In 2005, Sato et al. first identified anti-MDA5 in their Japanese cohorts of DM patients [25, 26]. In further studies, the link between anti-MDA5, muscle disease, lung disease, and peculiar skin abnormalities was reported [27].

In the reported clinical case, the patient presented, at the onset of the disease, Gottron's papules on her metacarpophalangeal and interphalangeal joints and elbows associated with periungual erythema and palmar papules. Gottron's papules are considered pathognomonic of DM, and they are more prevalent in anti-MDA5 seropositive patients than in seronegative ones [28, 29]. Other typical features in DM are heliotrope rash and a distinctive reddish or purplish rash over the face, neck, chest, shoulders, and mechanic's hands; they were absent in our patient. She also did not present cutaneous ulcerations that are considered prognostic negative factors associated with ILD progression [30].

Muscle involvement was present and mild; this could be considered another positive prognostic factor. Generally, it seems that MDA-5 antibody-positive patients who have coexistent muscle disease have a lower mortality rate than those without muscle involvement [31].

Finally, the patient presented objective evidence of lung involvement, as demonstrated by HRCT and spirometry, since the first months of the disease, and it worsened during the follow-up. However, she did not present respiratory insufficiency. The spectrum of ILD in anti-MDA5 DM can vary, and it is often severe, sometimes leading to intensive care unit admission and to death. Studies indicate that ILD progresses more rapidly in anti-MDA5-positive patients [9, 10]. This aspect has been of paramount importance in the management of the patient.

Glucocorticoids are the mainstay of the treatment for all IIMS; the rationale derives from old noncontrolled trials and case series, and they are commonly used in clinical practice [3, 12, 32]. Among immunosuppressive agents, mycophenolate mofetil has been reported as an effective therapeutic tool for autoimmune myopathies and, particularly, for CADM with lung involvement [33]; other authors have evidenced the effects of calcineurin inhibitors in IIMs and ILD [34]. The role of high-dose intravenous immunoglobulin *G* (IVIG) as a first-line therapy for lung and cutaneous manifestations in myopathies is still debated with controversial results from open studies, uncontrolled retrospective trials, and case reports [35]. Different recent studies have confirmed their efficacy and safety; however, considering the high cost, IVIG use is only recommended in refractory forms. According to literature evidence, we decided to treat our patient, after cyclosporine failure, with glucocorticoids and mycophenolate mofetil. We preferred to avoid cyclophosphamide and high-dose glucocorticoids because of their greater side effects. Unfortunately, lung function tests worsened in few months. Symptoms remained stable. After an interdisciplinary meeting with the pneumologist, we chose to treat our patient with RTX 1000 mg on two occasions 15 days apart, with careful monitoring of the disease course.

RTX, a chimeric monoclonal antibody for depleting B cells showing CD20 protein, seems effective for the treatment of a refractory skin and lung disease in IIMs [13–20]. RTX efficacy is supported by a randomised clinical trial realised in 2013, rituximab in myositis (RIM) [17], but also by further different case reports highlighting the efficacy of this drug in anti-MDA5 DM [18–20]. Autoreactive B cells have a key role in the pathogenesis of IIMs because they produce autoantibodies; moreover, they have a role in the secretion of inflammatory cytokines and in the activation of effector *T* cells. RTX could have a double positive effect in this process and could be indirectly implicated in preventing these immunological factors from attacking the target organ [36, 37].

After RTX, the patient presented a rapid improvement of clinical symptoms and cutaneous lesions; there was a reduction of inflammation indexes, and spirometry parameters evidenced a raise of DLco from 42% to 56% of the predicted value.

In our opinion, positive results in the treatment of the patient could derive from early diagnosis and aggressive therapy at initial stage of disease and, particularly, of ILD. The patient is currently under follow-up.

In conclusion, our case report confirms that anti-MDA5 DM is a severe autoimmune disease often associated with life-threatening ILD. Diagnosis can be difficult and defied with standard diagnostic studies and standard autoantibody searching. Antibodies to MDA5 have an important role in the diagnosis and in the phenotypical characterization of myositis, and they should be tested in the presence of clinical suggestive features. Outcome of the disease is poor, and it is of paramount importance to make a rapid diagnosis and to start as soon as possible with an aggressive therapy.

A defined therapeutic approach has not been identified. In our experience, RTX can be a valid therapeutic tool in the management of anti-MDA5 DM with both cutaneous and lung involvement, especially at the initial stage of the disease.

Further data are necessary to implement our knowledge of these pathologies, aiming to the best care and mortality reduction.
